# Screening indicators to evaluate the clinical significance of drug-drug interactions in polypharmacy among older adults with psychiatric disorders: a delphi study

**DOI:** 10.1186/s12888-024-05872-3

**Published:** 2024-06-04

**Authors:** Yu Liu, Xuefeng Li, Man Yang, Yaping Ding, Minghui Ji

**Affiliations:** 1 Department of Nursing, Kangda College of Nanjing Medical University, 88 Chunhui Road, Huaguoshan Avenue, Haizhou District, Lianyungang, Jiangsu Province 222000 China; 2 Department of Geriatrics, The First People’s Hospital of Lianyungang, No. 6 East Zhenhua Road, Haizhou District, Lianyungang, Jiangsu Province 222061 China; 3 Department of Psychiatry, The Fourth People’s Hospital of Lianyungang, Affiliated to Kangda College, Nanjing Medical University, No. 316 Jiefang East Road, Haizhou District, Lianyungang, Jiangsu Province 222000 China; 4 Department of Fundamental and Community Nursing, School of Nursing, Nanjing Medical University, 101 Longmian Avenue, Jiangning District, Nanjing, Jiangsu Province 211166 China

**Keywords:** Polypharmacy, Elderly, Psychotic disorders, Drug interaction

## Abstract

**Background:**

Polypharmacy is common in older adults with psychiatric disorders, but no consensus has reached about the reliable indicators evaluating the benefits and risks of drug-drug interactions (DDIs) in polypharmacy. We aimed to identify indicators suitable for evaluating the clinical significance of DDIs in polypharmacy in older adults with psychiatric disorders.

**Methods:**

The online tools were used to distribute and collect the questionnaires. The Delphi method was applied to analyze experts’ opinions. The degree of authority and coordination of experts were analyzed using the coefficient of variation, coefficient of coordination, expert’s judgment factor, familiarity with the study content factor, and Kendall coordination coefficient. Statistical analysis was conducted using the IBM SPSS® Statistics Package version 26.0.

**Results:**

After three rounds of expert consultation, five primary and eleven secondary indicators were identified. The primary “pharmacodynamic indicator” included “severity of adverse drug reactions”, “duration of adverse drug reaction”, “symptom relief”, “time to onset of symptomatic relief”, “number of days in hospital”, and “duration of medication”. The secondary “pharmacokinetic indicator” contained “dosage administered” and “dosing intervals”. The primary “patient tolerance indicator” contained one secondary indicator of “patient tolerability”. The primary indicator “patient adherence” contained one secondary indicator of “patient adherence to medication”. The primary indicator “cost of drug combination” contained one secondary indicator of “readmission”. These indicators were used to determine the clinical significance of DDIs during polypharmacy.

**Conclusions:**

The clinical significance of drug combinations should be taken into account when polypharmacy is used in the elderly. The five primary indicators and eleven secondary indicators might be preferred to evaluate their risks and benefits. Medication management in this population requires a multidisciplinary team, in which nurses play a key role. Future research should focus on how to establish efficient multidisciplinary team workflows and use functional factors to assess DDIs in polypharmacy for psychiatric disorders.

**Supplementary Information:**

The online version contains supplementary material available at 10.1186/s12888-024-05872-3.

## Background

New diagnostic techniques have uncovered a higher prevalence of psychiatric disorders among the elderly. Nagoor [1] conducted a community-based cross-sectional study, which revealed that 52.2% of individuals aged 60 years or above exhibited symptoms of psychiatric disorders. Similarly, Amoo [2] also confirmed a significant increase in psychiatric disorders among older adults. Although the prevalence of psychiatric disorders has not been studied systematically [3], Fischer [4] reported that the life-time prevalence of late-life psychotic disorders ranged from 0.3 to 1%. Older individuals with psychotic disorders may undergo acceleration of brain aging, indicating a cumulative biological effect of illness burden [5]. During the Covid-19 pandemic, psychosocial burden exhibits a rise initially, followed by a decline in patients with psychiatric disorders [6]. Older adults are at a higher risk of medication-related events, because of a higher physiological burden, polypharmacy for co-morbidities, or non-adherence to medications [7].

Polypharmacy is common in older adults with psychiatric disorders. Simultaneous use of five or more medications is widely accepted as polypharmacy [8]. The odd of polypharmacy is significantly higher in older adults with psychiatric disorders [9]. Antipsychotic polypharmacy occurred in 1 out of 20 older adults [10]. Lunghi [11] found that over a period from 2000 to 2016, the polypharmacy in the elderly with schizophrenia was mainly attributed to the overuse of non-antipsychotic medications, the DDIs in which might lead to serious adverse effects.

DDIs are frequent in polypharmacy [12], but many of them have just modest or no clinical significance [13, 14]. Wolff [15] reported that the risk of potential DDIs in older adults increased significantly with the number of additional drugs or the severity of diseases, and that patients with recurrent or severe depression were more likely to receive a combination of multiple antidepressant drugs. However, contemporary classifications for DDIs exhibit various loopholes [16], thus overestimating their clinical significances.

Clinical significance refers to the results could have genuine, palpable effects on patients’ health or on health care decisions made on their behalf [17] and the clinical significance of potential DDIs can be evaluated by minimal important change (MIC) [18]. However, there is a wide range of indicators to evaluate the clinical significance of DDIs, making it challenging to comparatively analyze the advantages and disadvantages of polypharmacy. In previous studies, the clinical significance of DDIs was mainly evaluated by efficacy and safety after medication [19]. Vazquez [20] investigated the clinical significance of DDIs from a pharmacokinetic perspective and Palleria [21] from pharmacokinetic and pharmacodynamic perspective. de Leon [22] discussed the clinical significance of DDIs in preventing adverse drug reactions (ADRs). Chippa [23] evaluated the clinical significance of DDIs according to their effects on the disability, falls, frailty, higher healthcare utilization, postoperative complications, mortality, and caregiver burden in older adults.

The aim of this study was to identify suitable indicators for evaluating the clinical significance of DDIs in polypharmacy in treating psychiatric disorders in the elderly.

## Methods

The Delphi method is widely used to explore for evaluation indicators. This study consisted of four phases: (1) a literature review, identification of indicators and development of questionnaires; (2) the first round of expert consultation, followed by a modification of the questionnaire; (3) the second round of expert consultation and analysis; and (4) the third round of expert consultation and identification of evaluation indicators.

### Identify items

Phase 1 involved a literature review of pharmacotherapy for older adults with psychiatric disorders. Included was a trial (1) randomized controlled or quasi-randomized controlled; (2) conducted on older adults; (3) comparing different drug treatments; (4) written in either Chinese or English; (5) published from Jun 1st 2013 to Jun 30th 2023. Evaluation indicators set in these trials were recorded and screened for the development of the first version of questionnaire.

Nineteen indicators were included and categorized into six dimensions, namely primary indicators. Six primary indicators and nineteen secondary indicators were selected into the questionnaire. Primary indicators were evaluated for the suitability of the setting. The importance of secondary indicators was assigned with a value from 0 to 4, with 0–2 indicating a relatively low importance, 3–4 a relatively high importance and 4 very important.

In Phase 2, a total of 12 experts were recruited to form an expert panel, which included psychiatrists, geriatricians, clinical pharmacists and nurse practitioners, with at least two in one field. Personal information of the experts was collected to facilitate contact and consultation with the experts during the multiple rounds of consultation. However, experts’ information was confidential and not reported to specific respondents. To reduce the variations in evaluation results, we required each expert to complete the questionnaire independently. Included were experts who (1) worked in a tertiary general hospital or a mental health center; (2) had worked for at least 10 years; and (3) had gained an intermediate or a senior title. One researcher contacted the staff at three hospitals, and staff at each hospital recommended 3–4 experts from their hospitals taken into account their curriculum vitaes and included criteria.

### First-round survey

The questionnaire was sent to the experts by Wechat app or E-mail and should be responded within two weeks. Experts’ personal information and suggestions for questionnaire indicators were compiled and discussed. Following that, indicators in the questionnaire were modified in accordance with the recommendations of experts. The primary indicators approved by the vast majority of experts based on the reasonableness were retained, and the secondary indicators that had been improperly set were excluded.

### Second-round survey

In the second-round survey, the data were collected using the same methodology as that in the first round. The parameters of each indicator were counted after the questionnaires were collected. The research team discussed the parameters and modified the indicators based on experts’ suggestions.

### Third-round survey

We re-sent the adjusted questionnaire to experts, and the final Kendall coefficient of each expert’s feedback was close to 1, indicating a strong consistency between experts’ responses. Finally, five primary and eleven secondary indicators were selected for the evaluation of polypharmacy.

### Statistical analysis

Data were compiled in WPS Office Excel. Experts’ agreement on clinical significance was examined using the coefficient of variation (CV) and the coefficient of coordination (CC). The Ca coefficient (expert’s judgment factor) and the Cs coefficient (the familiarity with the study content factor) were calculated. As with the content, the expert was assigned as highly familiar 0.9, relatively familiar 0.7, generally familiar 0.5, not very familiar 0.3, and not familiar 0.1. The weights for theoretical analysis (large 0.3, medium 0.2, small 0.1), practical experience (large 0.5, medium 0.4, small 0.3), reading literature (large 0.1, medium 0.1, small 0.1), and intuitive judgment (large 0.1, medium 0.1, small 0.1) were assigned. Ca and Cs were summed and averaged into an authority coefficient (Cr). CV = standard deviation/mean. A CV < 0.25 was considered as a consensus reaching among experts. The Kendall coordination coefficient was used to evaluate CC. A larger Kendall coordination coefficient indicated a higher degree of coordination. Statistical analysis was conducted using IBM SPSS® Statistics Package version 26.0.

## Results

### Information of experts

Twelve experts from Lianyungang and Nantong in Jiangsu Province participated in the first-round consultation, and one geriatrician of them withdrew from the second- and third- round consultations, due to his lack of familiarity with the topic (Table 1).

**Table 1:** Basic information of experts in round 1

Item	Category	Number of experts	%
Sex	Male	3	25.0
	Female	9	75.0
Age	30–40 years old	7	58.3
	40–50 years old	4	33.3
	50–60 years old	1	8.3
Profession	Psychiatrist	4	33.3
	Geriatrician	3	25.0
	Clinical pharmacist	3	25.0
	Nurse practitioner	2	16.7
Title	Intermediate title	3	25.0
	Senior title	9	75.0
Working years	10–20 years	10	83.3
	20–30 years	1	8.3
	30–40 years	1	8.3
Highest degree	Bachelor	7	58.3
	Graduate	5	41.7

### Active degree of experts

The recovery rates were all 100% in the three rounds of survey. In the first-round consultation, one expert suggested to add a secondary indicator. The active degrees of experts are listed in Table 2.

**Table 2:** The active degree of experts

Round	Number of questionnaires issued	Number of questionnaires collected	Effective rate of questionnaire recovery (%)	Rate of submission of questionnaire opinions (%)
1	12	12	100	8.3
2	11	11	100	0
3	11	11	100	0

### Authority degree of experts

The majority of experts (8/12, 66.7%) relied heavily on practical experience to evaluate the indicators. Five experts were relatively and six experts were generally familiar with the topic. The authority coefficients are listed in Table 3.

**Table 3:** The authority degree of experts

Round	Ca	Cs	Cr
1	0.91	0.57	0.74
2	0.91	0.59	0.75
3	0.91	0.59	0.75

### Coordination degree of experts

The CVs for primary and secondary indicators in the three rounds of expert consultation are listed in Table 4.

**Table 4:** The CV of each indicator in the three rounds of consultations

Round	Primary indicator	CV	Secondary indicator	CV
1	Pharmacodynamic indicator	0.00	Occurrence of adverse drug reactions	0.16
			Severity of adverse drug reactions	0.19
			Duration of adverse drug reactions	0.25
	Pharmacokinetic indicator	0.45	Plasma drug concentration	0.36
			Serum drug concentration	0.31
			Dosing interval	0.23
	Therapeutic efficacy	0.00	Symptom relief degree	0.22
			Time when symptoms begin to resolve	0.24
			Symptom recovery time	0.22
			Length of hospitalization	0.27
	Patient acceptance	0.30	Patient tolerance	0.19
			Patient adherence to medication	0.20
	Patient self-perception	0.85	Quality of life	0.23
			Self-care ability	0.31
			Basic motor ability	0.36
			Mental state	0.28
			Perception and social participation	0.31
	Cost-effectiveness	0.45	Cost of the patient’s medicine	0.37
			Cost of hospitalization	0.36
2	Pharmacodynamic indicator	0.00	Occurrence of adverse drug reactions	0.29
			Severity of adverse drug reactions	0.26
			Duration of adverse drug reactions	0.19
	Pharmacokinetic indicator	0.32	Plasma/serum drug concentration	0.26
			Dosing interval	0.32
	Therapeutic efficacy	0.00	Symptom relief degree	0.26
			Time when symptoms begin to resolve	0.18
			Symptom recovery time	0.14
			Length of hospitalization	0.29
			Length of administration	0.20
	Patient acceptance	0.76	Patient tolerance	0.27
			Patient adherence to medication	0.18
	Cost-effectiveness	0.00	Annual drug cost	0.24
			Annual frequency of adverse reactions	0.27
			Annual admissions	0.24
3	Pharmacodynamic indicator	0.00	Type of adverse drug reaction	0.32
			Severity of adverse drug reactions	0.14
			Duration of adverse drug reaction	0.25
			Symptom relief	0.12
			Time to onset of symptomatic relief	0.31
			Time to recovery of symptoms	0.32
			Number of days in hospital	0.27
			Duration of medication	0.16
	Pharmacokinetic indicator	0.32	Dosage administered	0.20
			Dosing intervals	0.27
	Patient tolerance	0.00	Patient tolerability	0.20
	Patient adherence	0.32	Patient adherence to medication	0.23
	Cost of drug combination	0.76	Cost of medication during hospitalization	0.38
			Readmission	0.20

In the first round, the experts showed a unanimous opinion on the “pharmacodynamic indicator” and “therapeutic efficacy indicator”, recognizing the relatively high importance of both the primary indicators and the majority of secondary indicators. However, the CVs for primary indicators, such as “pharmacokinetic indicator”, “patient acceptance indicator”, “patient self-perception indicator”, and “the cost-effectiveness indicator”, were all greater than 0.25, implying a disagreement among experts. The majority of experts considered that the primary indicators were appropriate, such as “pharmacokinetic indicator” (10/12, 83.33%), “patient acceptance indicator” (11/12, 91.67%), “patient self-perception indicator” (7/12, 58.33%), and “the cost-effectiveness indicator” (10/12, 83.33%).

The majority of experts assigned relatively high importance to “duration of adverse drug reactions indicator” (11/12, 91.67%), “plasma drug concentration indicator” (10/12, 83.33%), “serum drug concentration indicator” (9/12, 75.00%), and “the length of hospitalization indicator” (10/12, 83.33%).

Most CVs for secondary indicators of patient self-perception were greater than 0.25. Relatively low importance was assigned to “self-care ability indicator” (7/12, 58.33%), “basic motor ability indicator” (7/12, 58.33%), and “perception and social participation indicator” (7/12, 58.33%). Relatively high importance was assigned to “quality of life indicator” (7/12, 58.33%) and “mental state indicator” (8/12, 66.67%). Relatively low importance was assigned to “cost of the patient’s medicine indicator” (7/12, 58.33%) and “cost of hospitalization indicator” (8/12, 66.67%).

Based on the CVs of indicators and opinions of experts, “pharmacokinetic indicator”, “patient acceptance indicator”, and “cost-effectiveness indicator” were retained as primary indicators. “The patient acceptance indicator” and its secondary indicators, such as “self-care ability indicator”, “basic motor ability indicator”, and “perception and social participation indicator”, were deleted based on the CVs of indicators and opinions of experts. “Quality of life indicator” was deleted because it was overloaded with factors, and “mental state indicator” was deleted because it could be reflected by “therapeutic effect indicator”. A secondary indicator of “length of administration” was added. Considering of the diversity of drugs and the possibility that anticoagulants in blood collection tubes may affect some drug concentrations, “plasma drug concentration indicator” and “serum drug concentration indicator” were combined into one indicator. Based on experts’ opinion that two secondary indicators of “cost-effectiveness indicator” were relatively less important, they were replaced with three indicators: “annual drug cost indicator”, “annual frequency of adverse reactions indicator” and “annual readmission indicator”.

In the second round of consultation, the eleven experts demonstrated a unanimous opinion on the relatively high importance of “pharmacodynamic indicator”, “therapeutic efficacy indicator”, and “cost-effectiveness indicator”. However, the CVs for primary indicators such as “pharmacokinetic indicator” and “patient acceptance indicator” were greater than 0.25, which suggested a disagreement among experts. Ten experts (10/11, 90.91%) considered “pharmacokinetic indicator” and seven experts (7/11, 63.64%) considered “patient acceptance indicator” was appropriate.

Relatively high importance was assigned to “occurrence of adverse drug reactions indicator” (9/11, 81.82%), “severity of adverse drug reactions indicator” (8/11, 72.73%), “plasma/serum drug concentration indicator” (8/11, 72.73%), “dosing interval indicator” (8/11, 72.73%), “symptom relief degree indicator” (8/11, 72.73%), “length of hospitalization indicator” (9/11, 81.82%), “patient tolerance indicator” (9/11, 81.82%) and “annual frequency of adverse reactions indicator” (7/11, 63.64%).

Based on the CVs of indicators and opinions of experts, all primary indicators and their secondary indicators, except for “cost-effectiveness indicator”, were retained. Since the therapeutic effects and adverse reactions of drugs were about the pharmacodynamics of drugs, the primary indicator of “therapeutic effect indicator” was deleted and its secondary indicators were merged into one “pharmacodynamics indicator”. To manifest the differences in pharmacokinetic indicators, “plasma/serum drug concentration indicator” as a secondary indicator of “pharmacokinetic indicator”, was revised into “dose indicator”. Since “patient tolerance indicator” reflected the state of reduced responsiveness of the host to drugs, it was not appropriate to combine it with “patient acceptance indicator”. Therefore, the primary indicator “patient acceptance indicator” was deleted, and two primary indicators, including “patient tolerance indicator” and “patient adherence to medication indicator”, were added. The experts agreed that “cost-effectiveness indicator” was appropriate, and that three secondary indicators, including “pharmacodynamic indicator”, “pharmacokinetic indicator” and “patient acceptance indicator”, could all be used as indicators of “effectiveness”, therefore, “cost-effectiveness indicator” was revised into “cost of drug combination indicator”, and the secondary indicator into “cost of hospitalization indicator” and “readmission indicator”. To reflect the characteristics of drug combination, the descriptions of the secondary indicators were revised to better show the difference between indicators for coadministration and non-coadministration.

In the third round, the experts unanimously assigned relatively high importance to “pharmacodynamic indicator” (11/11, 100%) and “patient tolerance indicator” (11/11, 100%). However, the CVs for primary indicators such as “pharmacokinetic indicator”, “patient tolerance indicator” and “the cost of drug combination indicator” were greater than 0.25, which suggested a disagreement among experts. Ten experts (90.91%) considered “pharmacokinetic indicator” and “patient adherence indicator” and six experts (54.55%) considered “cost of coadministration indicator” was appropriate.

Relatively high importance was assigned to “severity of adverse drug reactions” (11/11, 100%), “symptom relief indicator” (11/11, 100%), “duration of medication” (8/11, 72.73%), “dosage administered” (8/11, 72.73%), “patient tolerability” (9/11, 81.82%), “patient adherence” (8/11, 72.73%), and “readmission” (9/11, 81.82%). However, fewer experts paid a relatively low importance to “type of adverse drug reaction” (6/11, 54.55%), “time to recovery of symptoms” (6/11, 54.55%), and “cost of medication during hospitalization” (7/11, 63.64%).

### Kendall coordination coefficient of indicators

The Kendall coordination coefficient of indicators in the three rounds of consultations are listed in Table 5. The overall Kendall coefficient was close to 1, indicating a high degree of consistency among experts.

**Table 5:** The Kendall coordination coefficient of indicators in the three rounds of consultations

Round	primary indicators		secondary indicators				total indicators	
	Kendall	*p*		Kendall	*p*	Kendall		*p*
1	0.188	0.046		0.262	< 0.01	0.654		< 0.01
2	0.303	0.010		0.165	0.030	0.703		< 0.01
3	0.245	0.029		0.169	0.034	0.702		< 0.01

After three rounds of analysis, all the primary indicators were retained and three less important secondary indicators were deleted. Finally, five primary indicators and eleven secondary indicators were considered suitable for evaluating the clinical significance of polypharmacy in the elderly Chinese.

## Discussion

Polypharmacy among the elderly, especially those with psychiatric disorders [6], has become a great health concern [24] and may result in serious DDIs, adverse side effects and poor compliance [25]. However, not all DDIs in polypharmacy is harmful [26], and related research remains to be expanded [27]. Therefore, it is necessary to set up efficient indicators to evaluate the benefits and risks of DDIs in polypharmacy. In the present study, after three rounds of expert consultation, we identified five primary indicators and eleven secondary indicators to evaluate the clinical significance of DDIs in polypharmacy.

In this study, “pharmacodynamic indicator” and “patient tolerance” were approved as primary indicators by all experts, which is in line with the findings in most pharmacotherapy studies. This may be due to the fact that physicians or psychiatrists are accustomed to evaluating the benefits of polypharmacy from their own professional perspective. Tan [28] took hypoglycemia risk to evaluate the coadministration of co-trimoxazole with sulfonylureas. Lickliter [29] conducted a randomized, placebo-controlled study, in which safety and tolerance of subjects were adopted to evaluate the combination of drug doses.

In this study, most experts did not consider “cost of the patient’s medicine” and “cost of hospitalization” of “cost of drug combination” important in evaluating the clinical significance of DDIs in polypharmacy. It is inconsistent with other studies, probably due to the short period set in this study. Marson [30] took health economic outcomes, evaluated according to incremental costs and quality-adjusted life-years (QALYs), as secondary outcomes. Wang [31] took stable disease-medication adherent, stable disease-medication nonadherent, relapse with hospitalization, relapse with ambulatory care, and death states every 3 months for 5 years as the evaluation indicators for the cost-effectiveness.

Medication management facilitates the monitoring and adjustment of polypharmacy in older adults. Non-adherence to medications or discrepancies between prescribed and digested medications is prevalent among older adults and can lead to treatment failure, and a multidisciplinary approach is recommended to optimize medication management [7]. He [32] built a multidisciplinary team and found that a family-involved smart medication management system could effectively improve medication adherence, self-efficacy in medication use, medication knowledge assessment scores, and family support for older adults. Interventions helping older people to anticipate and respond to medication-related problems may reduce the risk of harm associated with polypharmacy [33]. Yang [34] created a nurse-led medication self-management program for older adults with multimorbidity. Ibrahim [35] found that reasonable deprescribing among older adults could be safe, feasible, well tolerated and can lead to important benefits.

Nursing plays an important role in clinical medication [36]. A safe medication cannot continue without the involvement of nurses [37]. Mardani [38] found that nurses should cooperate with physicians and pharmacists in “evaluation of medication history”, “identification of medication discrepancies”, and “joint role in medication reconciliation”. Cheng [24] proposed that nurses should be given a bigger role in evaluating polypharmacy and medication.

### Strengths and limitations

This study focuses on the clinical significance of DDIs in polypharmacy among older adults with psychiatric disorders, and screened out a bunch of indicators for evaluating the benefits and risks of DDIs in polypharmacy from the perspectives of caregivers and patients.

It has three limitations. First, this study adopted the Delphi method, and the data were subjective. Some experts may have responded by a superficial thinking and the importance of one indicator might have been differently rated by experts from different fields, which affects the ranking of its importance. Second, the interval between two rounds was long, and some experts might propose inconsistent evaluations. Finally, due to the information bias of the researchers in preparing the questionnaire, functional factors of medication management and adherence were not listed in questionnaire.

## Conclusion

The clinical significance of drug combinations should be taken into account when polypharmacy is used in the elderly. The five primary indicators and eleven secondary indicators, identified in the present study, might be preferred to evaluate their risks and benefits. The primary “pharmacodynamic indicator” included “severity of adverse drug reactions”, “duration of adverse drug reaction”, “symptom relief”, “time to onset of symptomatic relief”, “number of days in hospital”, and “duration of medication”. The secondary “pharmacokinetic indicator” contained “dosage administered” and “dosing intervals”. The primary “patient tolerance indicator” contained one secondary indicator of “patient tolerability”. The primary indicator “patient adherence” contained one secondary indicator of “patient adherence to medication”. The primary indicator “cost of drug combination” contained one secondary indicator of “readmission”. Medication management in this population requires a multidisciplinary team, in which nurses play an important role. Future research should focus on how to establish efficient multidisciplinary team workflows and use these functional factors to improve medication management and adherence.

### Electronic supplementary material

Below is the link to the electronic supplementary material.

Supplementary Material 1

## Data Availability

Data generated or analyzed enquiries of this study can be directed to the corresponding author.

## Abbreviations

DDIs: drug-drug interactions
MIC: minimal important change
ADRs: adverse drug reactions
CV: coefficient of variation
CC: coefficient of coordination
Cr: authority coefficient
